# *Listeria monocytogenes* in Jiaxing: Whole-Genome Sequencing Reveals New Threats to Public Health

**DOI:** 10.3390/pathogens15010109

**Published:** 2026-01-19

**Authors:** Lei Gao, Wenjie Gao, Ping Li, Miaomiao Jia, Xuejuan Liu, Peiyan He, Henghui Wang, Yong Yan, Guoying Zhu

**Affiliations:** Jiaxing Key Laboratory of Pathogenic Microbiology, Jiaxing Center for Disease Control and Prevention, Jiaxing 314050, China; shuangyuzuo007@126.com (L.G.); gwjof2015@163.com (W.G.); plijxcdc@163.com (P.L.); jmm0649@163.com (M.J.); liuxuejuan@foxmail.com (X.L.); hepeiyan@aliyun.com (P.H.); jxcdc2010@126.com (H.W.)

**Keywords:** *Listeria monocytogenes*, whole-genome sequencing, virulence factor, antimicrobial resistance, serotypes

## Abstract

(1) Background: *Listeria monocytogenes* (Lm) is recognized by the World Health Organization (WHO) as one of the four principal foodborne pathogens. This study aimed to investigate the molecular characteristics of Lm isolates from Jiaxing, China, using whole-genome sequencing (WGS) to enhance our understanding of their molecular epidemiology. (2) Methods: A total of 39 foodborne Lm isolates and 7 clinical Lm isolates were analyzed via WGS to identify resistance genes, virulence factors, lineage, sequence type (ST), and clonal complex (CC). Antibiotic susceptibility was assessed using Minimum Inhibitory Concentration (MIC) testing, and serotypes were confirmed via multiplex PCR. (3) Results: We found that 39 food isolates were mainly lineage II (66.67%), with 13 STs; ST8 was the dominant ST, and 2 new types, ST3210 and ST3405, were found. Among the seven clinical isolates, lineage I was dominant (57.14%), and ST87 was the dominant ST. Serotype 1/2a was dominant, accounting for 54.35%, followed by 1/2b, which accounted for 36.96%. The overall antimicrobial resistance rate was 13.04%, with a multidrug resistance rate of 2.17%. All strains harbored LIPI-1 and LIPI-2, and five strains carried LIPI-3 genes: one strain belonged to ST619 of lineage I, two strains belonged to ST224 of lineage I, and two strains belonged to ST11 of lineage II. (4) Conclusions: This study clarified the genotype and serotype characteristics of *Listeria monocytogenes* in Jiaxing, as well as their molecular characteristics relating to drug resistance and virulence, thus providing a technical basis for improving exposure risk assessment of *Listeria monocytogenes*. Continuous monitoring, prevention, and control are recommended to further improve regional public health and safety.

## 1. Introduction

*Listeria monocytogenes* (Lm) is a Gram-positive, non-spore-forming, facultative anaerobic, and intracellular pathogen that can grow at low temperatures and is widely distributed in nature. Lm is commonly found in various food products, particularly raw and cooked meats, with mortality rates from outbreaks linked to meat products ranging from 15% to 67% [1]. The bacterium can be transmitted through the foodborne route, whereby humans or animals consume food contaminated with Lm. Infection with Lm may lead to listeriosis, which is an infectious diseases causing significant clinical harm. In recent years, there have been multiple reports of listeriosis outbreaks caused by the contamination of food with *Lm* in several countries [2,3]. It is believed that 99% of *Lm* infections are foodborne [4]. In 2023, listeriosis had the highest mortality and hospitalization rates among the foodborne diseases reported by the European Union, making it the most serious foodborne disease [5]. The mortality rate (CFR) of listeriosis is approximately 20–30% [6]. The United States reports approximately 1500 cases of listeriosis annually, accounting for only 0.02% of major foodborne pathogen infections, but these lead to 4% of hospitalizations and 19% of deaths [7]. The prevalence rate of *Lm* in dairy products in Samsen Province, Türkiye, is 3.7% [8], while in Italian poultry production, the rate is 26.7% [9], which has aroused widespread global concern. From 1964 to 2010, 28 provinces in China reported a total of 82 epidemic-related cases, 147 clinical cases, and 479 isolates of *Lm* [10]. There are reports of 562 cases of *Lm* in mainland China from 2011 to 2017, indicating a significant increase in the number of patients over the past decade [11]. This pathogen has therefore become a serious public health problem in China.

*Lm* includes four phylogenetic lineages (lineage I, II, III, and the rare lineage IV). Most cases of human listeriosis are associated with lineage I *Lm*, while lineage II *Lm* is commonly found in natural and farm environments [12,13]. Lineages III and IV are relatively rare and often isolated from ruminants [4]. This bacterium can be divided into 14 serotypes and 5 major molecular serogroups (IIa, IIb, IIc, IVa, and IVb), among which serotypes 1/2a, 1/2b, 1/2c, and 4b account for over 95% of clinical cases [14]. Lm relies on virulence factors to invade host cells and is primarily mediated by pathogenicity islands such as LIPI-1, LIPI-2, LIPI-3, and LIPI-4 [15,16,17], in that Lm is associated with adhesion, invasion, and colonization in host cells. LIPI-1 encodes virulence factors *InlA*, *InlB*, and Listeria hemolysin O, which are crucial for the intracellular life cycle of *Lm*. LIPI-2 encodes *ActA* protein, which enables bacteria to move within host cells [13]. The distribution of LIPI-3 and LIPI-4 is significantly correlated with the high virulence of certain strains of *Lm* [18]. LIPI-3 carries the lls genes (*llSA*, *llSB*, *llSD*, *llSG*, *llSH*, *llSP*, *llsX*, and *llSY*), encoding Listeria hemolysin S, which has hemolysis and cytotoxicity and can exert bactericidal effects and alter the host microbiota during infection [19]. LIPI-4 is a specific fiber disaccharide-family phosphotransferase system [20], which has been shown to be closely associated with placental and central nervous system infections [21].

Since the first isolation of antibiotic-resistant *Lm* in 1988, its drug resistance has become an increasingly serious problem [22]. Multidrug-resistant *Lm* strains have been isolated from food and the environment [23], and the presence of multidrug-resistant (MDR) pathogens in food has become an increasingly concerning public health issue worldwide [24]. It has been reported that the emergence of strains resistant to first- and second-line antibiotics in China (penicillin ampicillin and erythromycin) has posed a significant challenge for clinical drug selection [25]. Monitoring the antimicrobial sensitivity of *Lm* is crucial for tracking the development of drug resistance.

To better understand the prevalence and potential risk of Lm in Jiaxing, this study analyzed 39 foodborne isolates and 7 clinical isolates of Lm in Jiaxing from 2023 to 2024 to elucidate their serotypes, STs, virulence factors, and antimicrobial resistance patterns. This study aims to enrich the molecular diversity data on Lm strains and provide scientific support for food safety risk assessment and clinical prevention strategies, thus enhancing the capacity for early warning and responses to public health issues caused by Lm.

## 2. Materials and Methods

### 2.1. Source of Strains

Samples of foodborne strains were collected from 908 food samples in 15 different categories in Jiaxing City to carry out food risk monitoring, according to the “National Food Pollution and Harmful Factors Risk Monitoring Manual (2023 and 2024)”. According to the instructions in this manual, 25 g (mL) of the sample was added to 225 mL of LB1 enrichment solution (Hopebio, Qingdao, China) at 30 °C ± 1 °C for 24 h. Then, 0.1 mL was transferred to 10 mL of LB2 enrichment solution (Hopebio, Qingdao China) at 30 °C ± 1 °C for 24 h. The Lb2 secondary enrichment solution was inoculated in the Listeria chromogenic medium (Hopebio, Qingdao, China) and cultured at 30 °C ± 1 °C for 48 h. Suspicious colonies (blue and surrounded by an opaque ring) were picked out and identified using a mass spectrometer (Brooke, Bremen, Germany). A total of 39 Lm-positive strains were isolated and detected in samples from the city, which were sent to the microbiology laboratory of Jiaxing Center for Disease Control and Prevention by the disease control departments of various counties and cities. Clinical isolates were derived from human cases. According to the active monitoring program of foodborne pathogens in Zhejiang Province (2023 and 2024), human strains were obtained from 7 Lm isolates collected from serum, feces, and vomit samples of patients with acute gastroenteritis in Jiaxing maternal and child health hospital. The identity of all strains was confirmed by the Microbiology Department of Jiaxing Center for Disease Control and Prevention using matrix-assisted laser desorption/ionization time-of-flight (MALDI-TOF) mass spectrometry (Bruker, Bremen, Germany).

### 2.2. Main Instruments and Reagents

Key instruments and reagents included the VITEK 2 Compact fully automated microbial identification and antimicrobial susceptibility testing system (BioMérieux, Craponne, France), a MALD-TOF MS instrument (Bruker, Bremen, Germany), the Illumina NextSeq 550 sequencing system (Illumina, San Diego, CA, USA), a Gram-positive antimicrobial susceptibility testing plate (Thermo Fisher, East Grinstead, UK), a bacterial susceptibility test strip (E-test) with Meropenem, a bacterial susceptibility test strip (E-test) with trimethoprim–sulfamethoxazole (Kangtai, Wenzhou, China), and an *Lm* serotyping nucleic acid multiplex real-time fluorescence PCR kit (XABT, Beijing, China).

### 2.3. Whole-Genome Sequencing

*Lm* strains, preserved with magnetic beads, were inoculated onto blood plates and incubated at 37 °C overnight. The colonies of *Lm* from the blood agar plate were picked, and the total DNA of the collected Lm was extracted by the magnetic bead method, according to the manufacturer’s instructions (Qiagen, Hilden, Germany). DNA purity was assessed using a fluorescence photometer (CLUBIO, Taiwan, China), with absorbance ratios (A260 nm/A280 nm) ranging from 1.8 to 2.0. Library preparation was performed using the NEB Next Ultra DNA Library Prep Kit for Illumina (Illumina, San Diego, CA, USA), followed by whole-genome sequencing on the Illumina NextSeq 550 sequencing system. Quality requirements for sequencing data included genome coverage ≥ 95%; gene region coverage ≥ 98%; overall coverage depth ≥ 100×; and base data quality value Q30 ≥ 85%. The sequencing results were spliced with SPAdes 3.6 software to obtain the optimal assembly results, while phylogenetic analysis was conducted using the MEGA 10.0.

### 2.4. Lineage, Multilocus Sequence Typing (MLST), Clonal Complexes (CCs), and Serotype

The sequence types (STs) were determined based on the whole-genome sequencing results for the strains using the MLST website (http://cge.food.dtu.dk/services/MLST/, accessed on 15 August 2025). The analyses of the CC type and lineage results were based on whole-genome sequencing of the strains, which were analyzed using the BIGSdb-LM database (http://bigsdb.pasteur.fr/listeria/, accessed on 15 August 2025). Serological typing was performed using the monocytic *Lm* serological typing PCR kit (XABT, Beijing, China).

### 2.5. Analysis of Virulence Factors

Virulence genes were analyzed using VirulenceFinder 2.0 (https://cge.food.dtu.dk/services/ToxFinder/, accessed on 17 August 2025), and the selected pathogenicity island included LIPI-1 (*prfA*, *plcA*, *hly*, *mpl*, *actA*, *plcB*), LIPI-2 (*inlA*, *inlB*, *inlC*, *inlJ*), and LIPI-3 (*lllsA*, *lllsB*, *lllsD*, *lllsG*, *lllsH*, *lllsP*, *lllsX*, *lllsY*). Additional virulence-associated loci (*bsh*, *intA*, *ami*, *fbpA*) were also analyzed, with a minimum nucleotide identity threshold of 80%.

### 2.6. Antibiotic Resistance Genes and Antimicrobial Susceptibility Testing of the Strain

ResFinder-4.5.0 Sever (http://cge.food.dtu.dk/services/ResFinder/, accessed on 19 August 2025) was used to retrieve resistance gene carriage. Penicillin, ampicillin, and erythromycin were tested for antimicrobial susceptibility using the Gram-positive antimicrobial susceptibility testing plate (Thermo Fisher, East Grinstead, UK). The results for trimethoprim–sulfamethoxazole and meropenem were obtained using the bacterial susceptibility test strip (Kangtai, Wenzhou, China). Interpreted in conjunction with the results of the CLSI M45-A3; Methods for Antimicrobial Dilution and Disk Susceptibility Testing of Infrequently Isolated or Fastidious Bacteria. Clinical and Laboratory Standards Institute: Wayne, United States, 2015, and EUCAST v15.0; Breakpoint tables for interpretation of MICs and zone diameters. European Committee on Antimicrobial Susceptibility Testing (EUCAST): Basel, Switzerland, 2025.

## 3. Results

### 3.1. Distribution of Lm Detected in Foods

**Figure 1:**
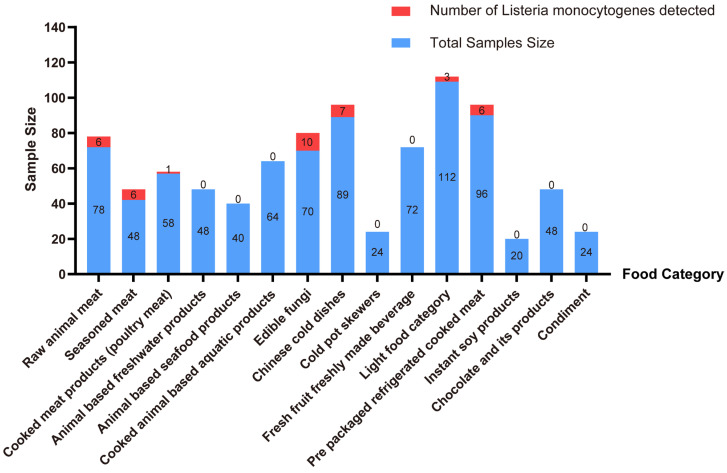
Detection of *L. monocytogenes* in different food sources.

A total of 908 food products were collected for the 2023 to 2024 national food safety risk monitoring in Jiaxing City, in which 39 strains of Lm were detected (4.30%). Among them, the isolation rates in seasoned meat (12.50%, 6/48), edible fungi (12.50%, 10/80), raw animal meat (10.41%, 5/48), and cold Chinese dishes (7.29%, 7/96) were relatively high (Figure 1).

### 3.2. Serotype Identification

**Figure 2:**
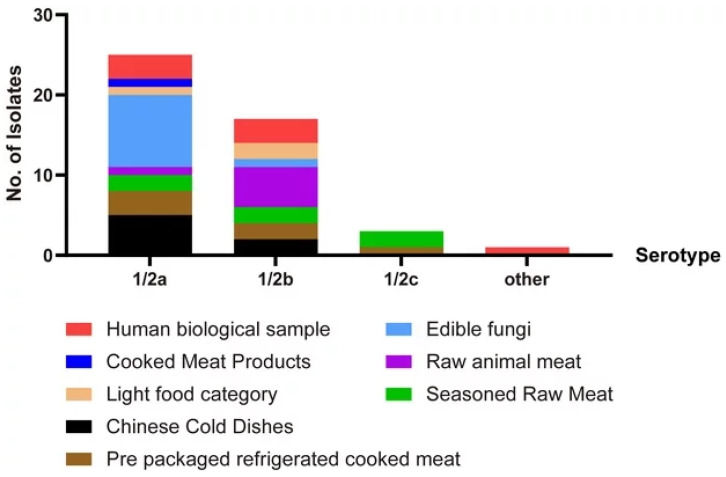
Schematic diagram of sample composition of different serotypes of *Listeria monocytogenes*.

PCR results were used to classify the 46 strains of Lm into four serotypes. The 1/2a serotype was dominant, with 25 strains (54.35%), followed by 17 strains of 1/2b (36.96%), 3 strains of 1/2c (6.52%), and 1 strain of other serotypes (2.17%). In addition, 1/2a and 1/2b were widely distributed (Figure 2).

### 3.3. Lineage, ST, and CC Distribution

Of the 46 Lm strains, 39 were from food isolates and 7 were from clinical isolates. Among the food isolates, lineage II accounted for 66.67%, and lineage I accounted for 33.33%. The bacterium was divided into 13 STs, with 13 strains of the ST8 type being the highest number, accounting for 33.33%; there were 5 strains of the ST87 type, accounting for 12.87%. Among them, strains LMJX2321 and LMJX2402 are newly applied sequence types (STs) by our center, via the website https://bigsdb.pasteur.fr/, accessed on 1 September 2025, with their corresponding numbers being ST3210 and ST3405, respectively. Among the seven clinical isolates, lineage I accounted for 57.14%, and lineage II accounted for 42.86%, while there were three STs and four ST87 strains, accounting for 57.14%. In total, 14 clonal complexes (CCs) were identified, with CC8 being the dominant group, accounting for 28.26% (Table 1).

### 3.4. Virulence Factors

**Figure 3:**
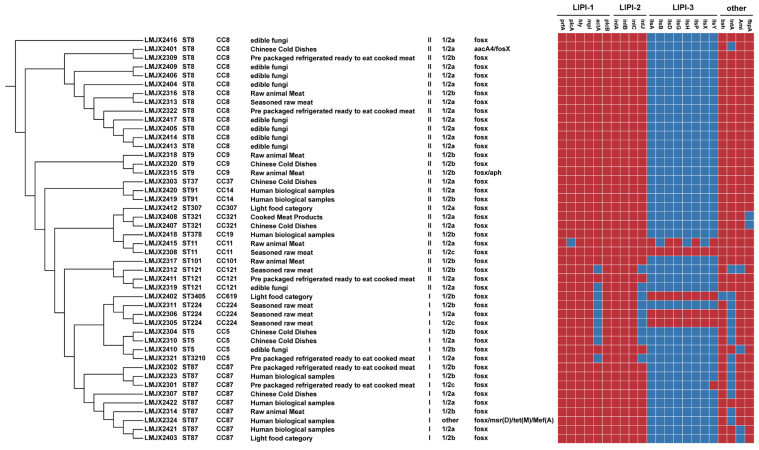
Phenotypic and genotypic characterization of Lm strains isolated from food and patients in Jiaxing City, China. Dendrogram of MLSTs, STs, CC types, PCR serotypes, antibiotic resistance profiles, and virulence factors were mapped using the ComplexHeatmap package. Legend: Red indicates the existence of the virulence gene, and blue indicates the deletion of the virulence gene.

The 46 strains from the *Lm* samples all contain LIPI-1 and LIPI-2, as shown in Figure 3. The positive rates of the *prfA*, *hly*, *mpl*, and *plcB* genes in LIPI-1, as well as the virulence genes *inlA*, *inlB*, and *inlC* related to LIPI-2, were all 100%. The *actA* gene of LIPI-1 and the *inlj* gene of LIPI-2 were deleted in 19.57% (9/46) of the isolates. Five strains carrying LIPI-3 were isolated from prepared meat, light food, and raw animal meat. One strain carrying part of the virulence gene of LIPI-3 belongs to ST619 and pedigree I. The carrying rate of virulence genes of two strains was 100%, belonging to ST224 of lineage I, while the other two strains also carried all eight virulence genes of LIPI-3, belonging to ST11 of lineage II.

### 3.5. Resistance Factors and Results of Antimicrobial Susceptibility Testing

In this study, all 46 strains carried *fos*X resistance genes, with LMJX2315 carrying both *aph* resistance genes and LMJX2324 carrying both *msr* (D), *tet* (M), and *Mef* (A) resistance genes. LMJX2401 also carried the *aaca4* resistance gene. All the above resistance genes were chromosomally located.

According to the standards of CLSI M45-a32015 and EUCAST, the overall drug resistance rate of the 46 strains was 13.04% (6/46). Resistance to penicillin and meropenem was observed in 2.17% (1/46) of isolates, resistance to ampicillin in 4.35% (2/46), and resistance to erythromycin and trimethoprim–sulfamethoxazole in 6.52% (3/46). Among the resistant isolates, four strains were resistant to a single antibiotic, one strain was resistant to two antibiotics, and one strain exhibited resistance to four antibiotics, qualifying as a multidrug-resistant strain. The multidrug resistance rate was therefore 2.17% (1/46) (Table 2).

## 4. Discussion

In this study, 908 food samples from different sources and ingredients in Jiaxing from 2023 to 2024 were detected. Of these, 39 samples were positive for *Lm*, representing an overall positive rate of 4.30%. The contamination rate of ready-to-eat food in Beijing is 7.11% [26], significantly higher than those of Heishan (0.7%) [27], Tokyo [28], Huzhou, Japan (2.4%) [29], and bulk retail food stores in Zhejiang Province (2.2%) [30]. This difference may be related to the sample size and composition, region, sanitary conditions, etc.

Lm is classified into 13 serotypes (1/2a, 1/2b, 1/2c, 4b, 3a, 3b, 3c, 4a, 4c, 4e, 4ab, 4d, and 7) based on serological reactions targeting flagellar (H) and somatic (O) antigens [31]. In the present study, the predominant serotypes of the 46 isolates were 1/2b, 1/2a, 1/2c, and others (except 1/2a, 1/2b, 1/2c, and 4b). These findings are consistent with the major serotypes that are reported to be prevalent in China [30,32,33,34]. However, unlike clinical isolates from countries such as France and Australia, where serotype 4b is the most prevalent [35,36], our study found 1/2a to be the dominant serotype, accounting for 54.53% of the total, followed by 1/2b, with 36.96%. Serotype 1/2a is widely distributed in various foods, which may be related to its more ready formation of biofilm and dominant position in the biofilm of mixed flora [37], making it more adaptable to different environments than other strains. Espinosa Mata et al. reported that the detection rate of serogroup 4B in *Lm* isolated from cheese was the highest [38]. In this study, the positive rate of 1/2a in edible fungi was as high as 90.9%, while the isolation rate of 1/2a in cold Chinese dishes was 71.4%, and the prevalence rate of 1/2b in raw meat was 83.33%. Whether there are specific dominant serotypes in different foods is worthy of further study. In this study, the serotype of one strain with other serotypes remains to be determined. In history, there have been numerous disease outbreaks caused by the change in the Lm serotype [2,39]. Relevant departments need to strengthen their monitoring to reduce the risk of foodborne listeriosis outbreaks.

According to genomic phylogeny, Lm can be classified into four lineages from I to IV. Among them, lineage I has a higher proportion of strains that were isolated from clinical cases and is associated with outbreaks of listeriosis, while lineage II is predominantly composed of strains isolated from food contamination and the environment [4,40]. The results of this study showed that lineage II was the main food isolate (66.67%), while lineage I was the dominant clinical isolate (57.14%), which was consistent with the conclusion of a previous study. In China, the most prevalent subtypes of Lm isolated from food sources are ST9 (29.1%), ST8 (10.7%), and ST87 (9.2%) [41,42,43]. The results of this study are slightly different: There are several STs in food isolates in Jiaxing, with ST8 (33.33%) and ST87 (12.82%) being the dominant STs. Studies have shown that the plasmid carried by ST8 helps improve its tolerance to high temperatures, salinity, acidic environments, oxidative stress, and disinfectants [44], in order to enhance its ability to become widely distributed and persist. In this study, 13 ST8 isolates accounted for 61.54% (8/13) of the edible fungi, which could be related to the acid–base environment and the impact of pollution on the soil in which common edible fungi are cultivated. In this study, the proportion of the ST87 genotype in clinical isolates was as high as 57.14% (4/7), and all these isolates came from critically ill patients, which was consistent with the main epidemic types of cases in China [30,45,46,47]. This suggests that the proportion of high-virulence epidemic strains of Lm in Jiaxing was relatively high. The proportion of ST87 in food isolates was only 12.82% (5/39), and there was a significant statistical difference in the distribution of ST87 between the two types of strains (*p* = 0.0166). It has been reported that the contaminated ST87 strain in ready-to-eat food is associated with clinical cases, suggesting that ST87 may be a high-risk genotype with strong pathogenicity and infection potential [48]. Four of the five ST87 strains that were isolated from food in this study came from chilled cooked meat, cold Chinese dishes, salads, and other ready-to-eat foods. Although no evidence of a direct correlation between ready-to-eat food and *Lm* in patients was found in this study, considering the dominant distribution of Lm ST87 in clinical isolates, it is still necessary to conduct in-depth research and monitoring at the molecular level for an extended period of time. ST3210 and ST3405 were both newly discovered in this study, which suggests that Lm in Jiaxing has undergone molecular-level variation and evolution, a phenomenon that warrants close monitoring by public health authorities.

The presence of virulence factors in Lm can reflect the different levels of harm caused by Lm strains. WGS allows for the identification of genes that are associated with high-pathogenicity islands. In the present study, 78.26% (36/46) of the isolates carried intact LIPI-1 and LIPI-2 virulence factors, suggesting that these virulence genes are relatively conserved in Lm. *act*A is critical for bacterial aggregation and biofilm formation [49], while the *inlJ* gene is directly involved in the passage through the intestinal barrier and the subsequent stages of infection [50] and a key virulence factor in Lm. In the present study, 19.57% (9/46) of the strains were deficient in both *act*A and *inl*J, indicating possible gene loss in less pathogenic strains. The *lls*X gene in LIPI-3 enhances the hemolytic activity and cytotoxicity of Lm, altering the host microbiota during infection [51]. Five strains in this study carried the virulence factor of LIPI-3 and were isolated from seasoned raw meat, light food, and raw animal meat. One of these belonged lineage I (ST619), while two belonged to lineage I (ST224), carrying all virulence genes of LIPI-3, which has previously been reported in a corresponding study in Zhejiang [30]. Another two strains were found to belong to lineage II (ST11), with full virulence factor carriage (100%). This challenges previous findings that LIPI-3 is exclusive to lineage I [4,25,46,52], suggesting changes in virulence gene profiles. Food safety monitoring should therefore be strengthened.

The overall drug resistance rate of the 46 isolates analyzed in this study was 13.04%. The most effective drugs for treating listeriosis are penicillin and ampicillin. The foodborne Lm isolate from Jiaxing showed resistance to first-line clinical drugs (penicillin, ampicillin, and erythromycin), and multiple drug-resistant strains appeared, which aligns with previous national and international studies [53,54,55,56]. A strain from a ready-to-eat Chinese salad is resistant to four drugs (AMP-ERY-SXT-MEM), and its potential risk should not be ignored. Another strain isolated from *Lm* patient serum was resistant to both ERY and SXT. This shows that multidrug resistance occurs not only in food, but also in patients. Although the sample size of this study is limited compared with a large number of domestic and international studies, the results are somewhat biased. However, the emergence of multidrug-resistant strains also calls for attention. Through the establishment of an accurate monitoring system, real-time mastery of antimicrobial resistance trends could be achieved, in order to provide a scientific basis for the rational use of clinical medication and effectively curb the dissemination and proliferation of antimicrobial-resistant strains to protect public health.

This study is based on the monitoring data for Lm in Jiaxing from 2023 to 2024. Due to the limitation of the overall sample size, our conclusions may have some limitations. Firstly, there are only seven clinical isolates in this study, and the sample size is small, which may lead to deviation in the results. In the future, long-term monitoring should be carried out to expand the sample size. At the same time, combined with the results of virulence gene expression detection (such as mRNA or protein level) and other experiments, our understanding of the high-virulence pathogenic mechanism and clinical harm of st87 and other genotypes was further improved. Secondly, as a part of the food risk monitoring program in Zhejiang Province, the insufficient sample size in some food sample categories (such as 20 ready-to-eat bean products, 24 cold pot noodles, and 24 condiments) also lead to estimation bias regarding the prevalence of Lm in these categories, making it difficult to objectively reflect the real pollution level. Finally, this study did not cover the entire food chain from raw material procurement, processing, and storage to sales in its sampling and could not locate the key link of pollution. For food categories with high positive rates, future studies may sample the entire food production chain or implement processing and sales links to clarify pollution links and subsequently employ more targeted prevention and control measures.

## 5. Conclusions

Our findings reveal that Lm in Jiaxing has undergone molecular-level variations, exhibited diverse serotype distributions, and shown changes in virulence factor profiles. The emergence of strains that are resistant to first-line therapies and multidrug-resistant variants highlights the need for increased vigilance and ongoing in-depth investigation by relevant authorities. In the future, long-term monitoring and further research are needed to comprehensively reveal the evolutionary law and pathogenic mechanism of this strain and provide a scientific basis for formulating accurate prevention and control strategies.

## Figures and Tables

**Table 1:** Distribution of Lm lineages, CCs, and STs of 46 strains.

Strain Category	Lineage	CC	ST	No. of Strains	Proportion of Category (%)	Proportion of Total Strains (%)
Food isolates (39 strains)	I			12	30.77	26.09
		CC5	ST5	3	7.69	6.52
			ST3210	1	2.56	2.17
		CC224	ST224	3	7.69	6.52
		CC87	ST87	3	7.69	6.52
		CC619	ST3405	1	2.56	2.17
	II			27	69.23	58.70
		CC8	ST8	13	33.33	28.26
		CC101	ST101	1	2.56	2.17
		CC121	ST121	3	7.69	6.52
		CC37	ST37	1	2.56	2.17
		CC11	ST11	2	5.13	4.35
		CC9	ST9	3	7.69	6.52
		CC321	ST321	2	5.13	4.35
		CC307	ST307	1	2.56	2.17
Clinical isolates (7 strains)	I			4	57.14	8.70
		CC87	ST87	4	57.14	8.70
	II			3	42.86	6.52
		CC19	ST378	1	14.29	2.17
		CC14	ST91	2	28.57	4.35
Total strains	I			16		34.78
	II			30		65.22
				46		100

**Table 2:** Resistance profile of Lm (*n* = 46).

No. of Antibiotic- Resistant Profiles	Antibiotic-Resistant Profile	No. of Strains	Strain Source	Percentage (%)
0		40	-	86.96
1		4		8.70
	AMP	1	Duck wing	2.17
	ERY	1	Cold Chinese dishes	2.17
	SXT	1	Salad	2.17
	MEM	1	Edible fungi	2.17
2	ERY-SXT	1	Human serum	2.17
4	AMP-ERY-SXT-PEN	1	Cold Chinese Dishes	2.17

Legend: ^1^ “AMP”—ampicillin; “ERY”—erythromycin; “SXT”—trimethoprim–sulfamethoxazole; “MEM”—meropenem; “PEN”—penicillin. ^2^ “ERY-SXT” indicates simultaneous resistance to erythromycin and compound sulfamethoxazole, while “AMP-ERY-SXT-PEN” indicates simultaneous resistance to ampicillin, erythromycin, compound sulfamethoxazole, and penicillin. ^3^ “-” represents all 40 strains of bacteria except drug-resistant bacteria, including 5 strains from cold Chinese dishes, 1 strain from cooked meat products, 9 strains from edible fungi, 6 strains from human biological samples, 2 strains from the light food category, 5 strains from prepackaged and refrigerated ready-to-eat cooked meat, 6 strains from seasoned raw meat, and 6 strains from raw animal meat.

## Data Availability

The data used in this study are owned by the Jiaxing Center for Disease Control and Prevention and were made available for this research to support related management activities. These data can be requested from the corresponding authors. For inquiries, kindly direct requests to the Jiaxing Center for Disease Control and Prevention, through the corresponding (G.Z.) or first author (L.G.).
